# Electricity in Electro-Therapeutics

**Published:** 1897-03

**Authors:** 


### BOOK NOTICES.

ELECTRICITY IN ELECTRO-THEAUPEUTICS. By Edwin J. Houston, Ph. D., and A. E. Kennelly, Sc. D. New York : The W. J. Johnson Company, 253 Broadway. 1896. Cloth, 400 pages, 128 illustrations. Price, $1.00.

This little volume constitutes one member in a series entitled "Elementary Electro-technical Series," published by The W. J. Johnson Co., publishers of *The Electrical World*, the pioneer electrical journal of America.

The book under notice gives the latest and most approved views on electricity and electrical phenomena. Discarding the old idea of two kinds of electricity, positive and negative, it gives the most modern views of the nature of electricity and the phenomena of attraction and repulsion.

The principal attention of the authors has, however, been concentrated upon those forms of electrical energy which are produced by the voltaic cell and by the dynamo. The nature of the volt, Ohm, and ampère is briefly described, and diagrams giving the character of the electrical current from various kinds of dynamos are shown with a view of making clear the ideas involved in the numerous fluctuations of the currents arising from these machines.

No very detailed descriptions of the apparatus used are given, the object being to give clear general views of the laws of electricity and the circumstances under which they operate, that medical men may use electrical apparatus in their practice, with a clear idea of the principles underlying its structure and mode of action.

Nothing in the way of advice as to how to apply electricity in any given case is to be found in this book, and indeed should not be expected, since such information ought rather to be sought in books on the clinical applications of electricity. What this book does give, we repeat, is clear and deep understanding of the underlying principles in the construction and action of medical batteries. Copious illustrations complete these explanations, and make it a very desirable book for the physician who practices with electricity.

